# Interactions between the sexual identity of the nervous system and the social environment mediate lifespan in *Drosophila melanogaster*

**DOI:** 10.1098/rspb.2018.1450

**Published:** 2018-11-28

**Authors:** Ewan O. Flintham, Tomoyo Yoshida, Sophie Smith, Hania J. Pavlou, Stephen F. Goodwin, Pau Carazo, Stuart Wigby

**Affiliations:** 1 Edward Grey Institute, Department of Zoology, University of Oxford, Oxford OX1 5PS, UK; 2 Centre for Neural Circuits and Behaviour, University of Oxford, Oxford OX1 3SR, UK; 3 Cavanilles Institute of Biodiversity and Evolutionary Biology, University of Valencia, Valencia, Spain; 4 Department of Life Sciences, Imperial College London, Ascot, SL5 7PY, UK

**Keywords:** *Drosophila*, sexual selection, sexual conflict, life history, lifespan

## Abstract

Sex differences in lifespan are ubiquitous, but the underlying causal factors remain poorly understood. Inter- and intrasexual social interactions are well known to influence lifespan in many taxa, but it has proved challenging to separate the role of sex-specific behaviours from wider physiological differences between the sexes. To address this problem, we genetically manipulated the sexual identity of the nervous system—and hence sexual behaviour—in *Drosophila melanogaster*, and measured lifespan under varying social conditions. Consistent with previous studies, masculinization of the nervous system in females induced male-specific courtship behaviour and aggression, while nervous system feminization in males induced male–male courtship and reduced aggression. Control females outlived males, but masculinized female groups displayed male-like lifespans and male-like costs of group living. By varying the mixture of control and masculinized females within social groups, we show that male-specific behaviours are costly to recipients, even when received from females. However, consistent with recent findings, our data suggest courtship expression to be surprisingly low cost. Overall, our study indicates that nervous system-mediated expression of sex-specific behaviour *per se*—independent of wider physiological differences between the sexes, or the receipt of aggression or courtship—plays a limited role in mediating sex differences in lifespan.

## 1. Introduction

Sexual dimorphism in lifespan is widely observed in animals, including in our own species [1–3]. However, while sex differences in lifespan are widespread, patterns vary considerably between taxa; for instance, most mammals show male-biased mortality, with the opposite being generally true of birds [4,5], while in insects male-biased mortality has been reported for some species, but there remains little certainty as to any broad taxonomic patterns (e.g. [6,7]). This diversity in patterns of sex-dependent life expectancy, observed between and within taxonomic groups, has been linked with a number of non-exclusive factors, including variation in mating systems, genetic sex determination and social environments [8–10], but remains one of the most complex problems in ageing biology.

Models from life-history theory predict that variation in lifespan arises from the limited lifetime resource allocation that is available to individuals, as selection will favour an optimal resource distribution across somatic and reproductive functions, which maximizes overall organismal fitness [11]. Thus, if selection favours increased investment in mating and reproduction at the expense of somatic upkeep, an individual will age more rapidly [7,12,13]. Sex differences in senescence and lifespan could then result from divergent life-history strategies and subsequent resource distribution optima between the sexes. For instance, in cases of classic sex roles, male strategies are usually characterized as ‘high risk, high attrition’ while females are predicted to follow more conservative ‘low risk, low attrition’ strategies [14]. Simply put, males tend to gain high reproductive rewards from obtaining many matings, and thus certain risky behaviours—such as fighting rivals for mating territories—may be worthwhile. Meanwhile, lower potential payoffs to females for similar behaviours may select for more a more risk-averse, long-term strategy. Thus, overall, male-biased mortality would be expected [14]. However, more recent theory and empirical work indicate complex interrelationships between sexual selection, sexual conflict and life-history traits such as senescence. Therefore, disentangling the evolutionary mechanisms underpinning sex differences in ageing has proved difficult [5,14].

The influence of inter- and intrasexual social interactions on sex-specific lifespan patterns has been well documented in a wide range of taxa [15–19]. In particular, a body of work has emerged which is consistent with sex-specific behaviours underpinning the effects of social interactions on lifespan, due to the role behaviour can play in mediating energetic expenditure [5,20–31] (reviewed in [32]). However, evidence from recent studies in insects, which have attempted to explicitly partition behavioural from wider reproductive costs such as copulation, has challenged the idea of a trade-off between energetic expenditure associated with behaviour and survival [33–35]. Currently, the consequences of sex-specific behaviours for the lifespan of both actors and recipients remain unclear. Furthermore, even investigating the relationship between sex-specific behaviour and lifespan has proved challenging because it is difficult to separate the effects of behaviour from unrelated sex differences in physiology, such as hormone profiles or morphology, which are often present in concert [14].

Here, we sought to disentangle the role of expressing and receiving sex-specific behaviours, encoded by sex-specific neural circuitry, from wider physiological differences between the sexes, in mediating lifespan in *Drosophila melanogaster*. *Drosophila melanogaster* are polygynandrous, and males compete intensely for access to females. In many studies, males have been found to possess shorter lifespans than females, and it has been suggested that male-specific behaviour, such as courtship, may underpin sex-differentiated lifespan patterns in the species [20,32]. To investigate this, we first exploited cell autonomous sex differentiation in *D. melanogaster* to genetically manipulate the sexual identity of the nervous system (NS) [36,37]. We then observed behavioural and lifespan responses to varying social conditions intended to elicit different types of sex-specific behaviour. Inversing NS-identity induces asymmetric behavioural responses across the sexes. In females, NS-inversion (or ‘masculinization’) has previously been shown to engender male-specific behaviours including courtship of other females, while the inverse—feminization in males—is known to reduce sex discrimination, inducing courtship of both males and females at high rates and reduced intrasexual aggression [37,38].

## 2. Material and methods

### (a) Fly genetics, stocks and culture

The transgenic lines of used in all experiments were from Canton-Special (Canton-S) background. Some experiments also used flies from the Dahomey (Dah) stock, as specified below. We manipulated the sexual identity of the NS by suppressing *tra* expression in the female nervous system using RNAi, or by expressing the female isoform of *tra* in males. The *tra* switch is thought to influence the development of the sexually dimorphic mAL interneuron cluster via alternative splicing of *fruitless* primary transcripts, producing a male-like cluster in masculinized females and vice versa in feminized males [38]. There is no evidence to date to suggest that manipulations of *tra* affect pheromone profiles [37]. Masculinization has been shown to reduce female receptivity to mating by males [39], although it is unclear to what extent this is a result of a change in receptivity to male courtship, which is thought to be controlled independently of the *fruitless* cascade [40].

NS-inversion was achieved through crossing females carrying the nervous system-specific GAL4 driver (*elav-*GAL4) with UAS-*traIR* or UAS-*traF* males to produce masculinized females and feminized males, respectively. Control genotypes were the progeny of crosses between *elav-*GAL4, UAS-*traIR*, UAS-*traF* and Canton-S wild-type stock. A total of six genotypes were used in the experiments: (i) *elav-*GAL4/UAS-*traIR* (masculinized female), (ii) *elav-*GAL4/UAS-*traF* (feminized male), (iii) +/UAS-*traIR* (control-traIR females), (iv) +/UAS-*traF* (control-*traF* males), (v) *elav-*GAL4/+ (control-*elav* males and females) and (vi) +/+ (wild type).

All flies were maintained on a sugar-yeast molasses medium [41] and maintained at 25°C on a 12 L : 12 D cycle. Crosses were initially conducted in bottles after which females were placed in cages on an agar-grape juice medium, supplemented with live yeast; eggs laid on the medium were then placed at a density of 50 eggs per 7 ml vial using a standard density method [42]. Adult offspring were collected as virgins using ice anaesthesia within 8 h of eclosion. Experimental flies were transferred to fresh vials every 3 days using mild CO_2_ anaesthesia.

### (b) Experimental design

#### (i) Experiment 1: effects of nervous system identity and social interactions on behaviour and lifespan in males and females

**Figure 1.**
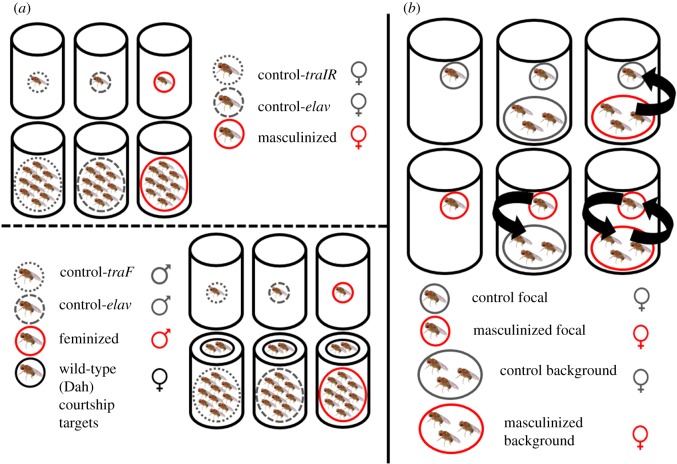
(*a*) Experimental design for Experiment 1. Control and NS-inversed (masculinized and feminized) male and females were housed either individually or in group treatments of 10 in vials. Two wild-type (Dah) females were added to male group treatments. (*b*) A scheme for the experimental design in Experiment 2. Control and focal flies were housed either individual or in group treatments of three wild-type or three masculinized background flies. Arrows indicate the expected direction of courtship behaviour. (Online version in colour.)

To investigate the effects of nervous system identity and social experience on behaviour and lifespan, masculinized, feminized and control flies were housed in vials either individually or in groups containing 10 flies (figure 1*a*). Comparisons between experimental and control individually housed flies allowed us to test the effect of NS-identity on lifespan in the absence of social interactions, which was required to control for any lifespan differences that occur due to effects of the genetic manipulations that are unrelated to the expression of social behaviour. Differences between group and individual treatments, meanwhile, would indicate an influence of the social environment. To ensure males in the grouped treatments had courtship targets, while ensuring that mating was rare, we added two mated wild-type Dah females to each vial (making 12 flies in total); these females were replaced weekly when the experimental flies were moved on to new food. When a vial in the male group treatments contained five or fewer experimental males, the number of wild-type females was reduced to one, in order to reduce sex ratio variability across replicate vials.

#### (ii) Experiment 2: effects of masculinization of the nervous system in females on the lifespan costs of expressing versus receiving male-like behaviour

In a second experiment, we focused on masculinized females to examine the lifespan costs of females expressing male-specific behaviour, and the costs to other females of receiving male-like behaviours—such as courtship or male-type aggression—from those masculinized females. Female flies (masculinized females and control females) were assigned to vials across nine treatment groups as part of a factorial design (figure 1*b*). Vials contained a focal female whose behaviour and lifespan were recorded. Focal females were either masculinized, or one of the two control genotypes, and were housed in one of three social environment treatments: (i) individually, (ii) alongside three non-focal (hereon ‘background’) non-masculinized (Canton-S wild-type or other controls) females or (iii) with three non-focal (background) masculinized females. The different treatment groups were designed to isolate distinct types of sexual behaviour, giving/receiving courtship and aggression, such that their independent effects on lifespan could be inferred. Masculinized focal females housed with non-masculinized background females should be involved in performing courtship but not receiving any in return, while the reverse should be true of control females in a masculinized environment. Control females held with non-masculinized background females should neither exhibit nor receive any courtship. Meanwhile, masculinized focal females held with other masculinized background females should be involved in the full range of courtship-related behaviours, both giving and receiving. To visually differentiate between focal and background flies, the wings of focal flies were clipped diagonally, approximately two-third of the way towards the tip. This procedure is not thought to have fitness or behavioural consequences [43]. Any dead background flies were replaced from a stock which was maintained throughout the experiment, and full replacement of all background flies occurred at three-week intervals to reduce co-ageing effects of the background flies.

### (c) Observation of behaviour and lifespan measurement

Deaths were recorded daily, and any escaped or accidently killed focal flies were right-censored in data analyses. We quantified aggression and courtship behaviours in grouped treatments, for both experiments. Mating, which was only possible in the grouped male treatments of the first experiments (where two females were provided as targets of courtship), was only seen once in the whole experiment and thus was not subsequently analysed. Observed aggression displays included ‘lunging’, ‘head butting’, ‘body shoving’ and ‘leg fencing’ in a low or high posture [44]. Observed courtship behaviours were ‘orientation’ of an individual's body axis towards a recipient, wing extension at 90°, ‘tapping’ of an initiator's foreleg on the thorax or another recipient, ‘singing’ through perpendicular wing extension by a focal fly and ‘licking’, whereby a fly follows closely behind another attempting to lick its genitalia and attempting copulation [38,45]. In treatments with multiple feminized males or masculinized females, a unique behaviour known as ‘chaining’ was observed, whereby flies form chains of three or more participants, with each individual initiating courtship with the fly in front and receiving courtship from the fly behind, interspersed with aggression [46,47]. In the first experiment, behavioural observations were conducted for masculinized, feminized and control flies housed in group treatments twice every week on separate days for 1–2 h over five weeks, except on the first week when only one observation was taken following fly maturation earlier in the week. During observation, vials were placed on racks and systematically scan-sampled for behaviours using a presence/absence recording rule for each behaviour (courtship and aggression) in each direction (male-to-female, male-to-male and female-to-female). For every vial, we calculated the cumulative totals for each behaviour-direction combination given by summing observed behaviour events across all systematic scans for each combination. This gave the total number of observations of each behaviour and non-behaviour for each vial, giving appropriate data for the GLMs used in the statistical analyses. A similar method was adopted in Experiment 2, with the only differences that observations occurred in the first three weeks alone, and only behaviour involving the vial's focal fly was recorded, as was the direction of courtship behaviour (i.e. whether it was given or received by the focal fly). In the occurrence of chaining, a score of 1 was given to all behaviours.

### (d) Statistical analysis

All statistical analyses were conducted in R v. 3.3.1. Backward model selection was used, with nonsignificant factors being sequentially removed. Male and female analyses were conducted independently, and aggression and courtship behaviour data were analysed separately. All analyses considered two factors: fly NS-identity (focal fly identity in Experiment 2) and Social Environment. Male control-*elav* and control-*traF* flies did not differ significantly in lifespan or behavioural observations (see following paragraphs for description of statistical tests) and so the data were pooled in to one ‘control’ treatment group to simplify analyses (electronic supplementary material, figures S1–S3). In Experiment 1, control-*traIR* females lived slightly longer than control-*elav* females. However, there was no interaction with social environment treatment in either experiment (Experiment 1: control genotype × group housing; *p* = 0.988; Experiment 2: control genotype × wild-type environment; *p* = 0.992, control genotype × masculinized environment; *p* = 0.768; electronic supplementary material, figures S4 and S5), and female control strains did not differ from each other behaviourally (electronic supplementary material, figures S1 and S2), so for consistency control data were pooled in to a combined female control group. After data pooling, NS-identity had two treatment levels: ‘control’ and ‘NS-inversed’, while social environment had two treatments in Experiment 1: ‘individual’ and ‘group housing’, and three treatments in Experiment 2: ‘individual’, ‘wild-type background’ or ‘masculinized background’.

When analysing courtship and aggression, we used GLMs with quasi-binomial error distribution to account for overdispersion. We modelled our response variable as the cumulative total of observations where a given behaviour occurred for each vial and the cumulative total of observations where the behaviour was not observed for each vial, using the cbind function in R. We modelled NS-identity as a fixed effect in Experiment 1, and focal NS-identity, social environment and their interaction as fixed effects in Experiment 2. As sexual behaviours were not observed in flies housed individually, we did not include Individual treatments in our behavioural analyses.

Lifespan data were analysed using the Survival Analysis package [48]. Unless specified, fly lifespan was analysed through a Cox proportional hazards model with censors. A global goodness-of-fit test using scaled Schoenfield residuals indicated that the assumptions of the proportional hazards model were violated by survival data of females in Experiment 1 (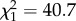, *p* = 1.06 × 10^−7^). As a result, we fitted a parametric survival regression with extreme value error distribution instead. We modelled NS-identity (or focal NS-identity in Experiment 2), social environment and their interaction as a fixed effect in analyses for both experiments. All *p*-values associated with a *χ*^2^ test statistic refer a likelihood ratio test for an interaction between factors, while *p*-values associated with a *z*-score refer to pairwise comparisons between different factor levels based on the ratio of model coefficient (e.g. hazard) estimates to their standard error. For lifespan data, the baseline treatment groups were control-individual in Experiment 1, and control focal—individual in Experiment 2.

## 3. Results and discussion

### (a) Male-like behaviour is associated with lifespan responses to group living

Our first aim was to assess the effects of masculinization and feminization on the expression of two important sexual behaviours: courtship and aggression, and secondly, to identify any relationships between sex-specific behavioural and lifespan patterns. In Experiment 1, we housed control and NS-inversed flies either individually or in groups, where sexual behaviours are expected to occur, and observed behavioural and lifespan patterns across treatment groups (figure 1).

**Figure 2.**
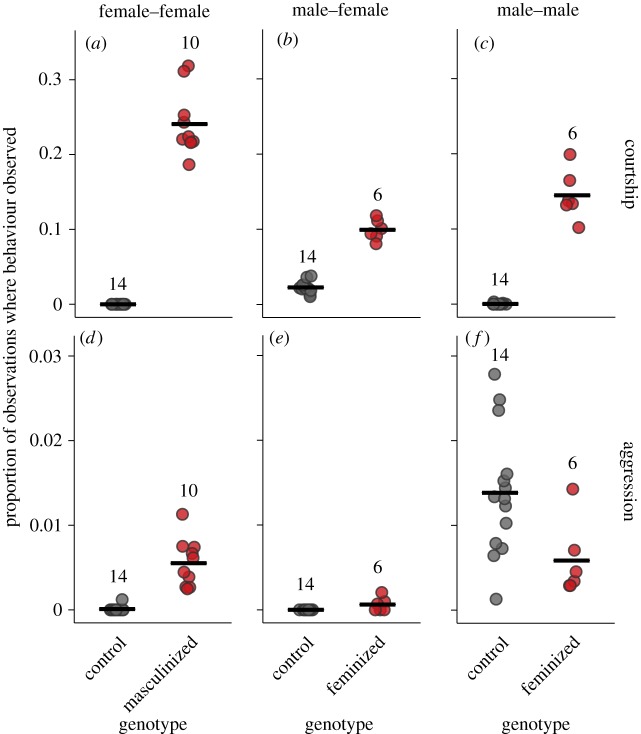
The effect of nervous system manipulation (masculinization and feminization) on behaviour in Experiment 1. Per vial cumulative proportions, mean averaged across the 10 flies in each vial, are shown for (*a*–*c*) observed courtship and (*d*–*f*) observed aggression. Horizontal black lines representing mean averages. Female–female and male–male interactions refer to intrasexual behaviour, while male–female interactions are intersexual behaviours performed by males on the two female ‘courtship target’ flies. Sample sizes shown refer to the number of independent vials observed. (Online version in colour.)

As expected, we found that manipulating NS-identity induced clear behavioural responses in males and females, but that these effects were not symmetrical across the sexes (figure 2). In females, masculinization induced male-like behaviour patterns by introducing intrasexual courtship (*t*_22_ = 9.333, *p* < 0.0001) and dramatically elevating intrasexual aggression (*t*_22_ = 8.224, *p* < 0.0001, 50-fold increase in mean behaviour rate). In males, however, NS-inversion does not produce a female-like behaviour profile. Instead, feminization appears to result in reduced sex discrimination, resulting in high rates of both intersexual courtship (*t*_18_ = 14.02, *p* < 0.0001, fourfold increase in mean behaviour rate) and aggression (*t*_18_ = 3.22, *p* < 0.005, intersexual aggression not observed in controls) compared with controls, as well as inducing intrasexual courtship (*t*_18_ = 14.02, *p* < 0.0001). Interestingly, however, intrasexual aggression appeared reduced in feminized males than in controls (*t*_18_ = −2.336, *p* = 0.031, 57.8% reduction in mean behaviour rate), a result which appears consistent with existing work, although the details of the neurogenetic mechanisms underlying this effect remain unclear [37].

**Figure 3.**
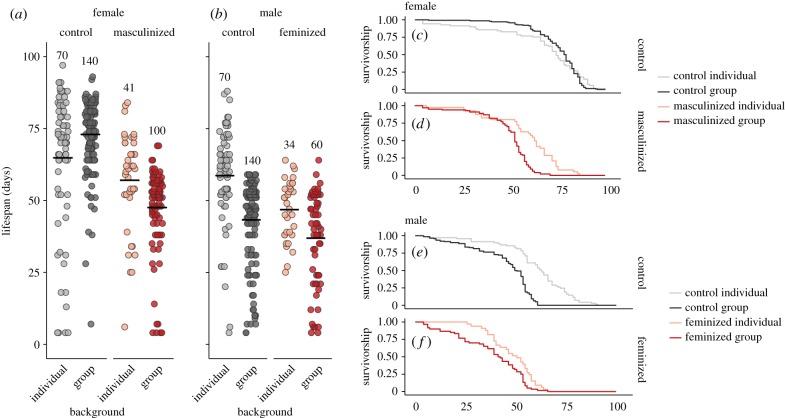
The effect of nervous system manipulation (masculinization and feminization) and social environment (individual versus group housing) on lifespan in days for females and males in Experiment 1. (*a*,*b*) Age at death of each focal female, with horizontal black lines representing mean averages. Sample sizes refer to the number of individual focal flies. (*c*–*f*) Survivorship curves, which give the proportion of survivors through time in days for each treatment. (Online version in colour.)

Both male (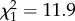, *p* = 0.0006) and female (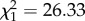, *p* < 0.0001) lifespan patterns were best explained by an interaction between NS-identity and social environment (figure 3). NS-inversion had clear independent lifespan costs in both sexes, as feminized (*z* = −5.47, *p* < 0.0001, 12.0% difference in mean lifespan) and masculinized (*z* = 5.761, *p* < 0.0001, 20.2% difference in mean lifespan) individuals lived less long than controls, indicating that there are potential health costs to a mismatched nervous system and body. However, NS-inversed and control flies showed different lifespan responses to group living. In females, grouped controls showed indistinguishable lifespan patterns to individuals (*z* = −0.33, *p* = 0.74), while masculinized flies showed a significant cost to being grouped (*z* = −6.84, *p* < 0.0001, 16.7% difference in mean lifespan). Importantly, the lifespan pattern observed in masculinized females appeared qualitatively very similar to that observed in control males, as they also suffered a clear lifespan reduction when living in groups (*z* = 8.39, *p* < 0.0001, 26.2% difference in mean lifespan), indicating that a male nervous system, or the male-like behaviour thereby induced, may be important in determining lifespan patterns across social environments. To statistically compare the similarity of the individual-group lifespan shifts encountered by masculinized females and control males, we ran a post hoc analysis using a cox proportional hazards model with two factors with two levels each: fly type (masculinized female versus control male) and social environment (individual versus group). As we already knew the direction of the lifespan response to grouping occurred in the same direction in both fly types (i.e. they both decreased), we were interested in the magnitude of the shift in both groups. We found that individual control males and masculinized females did not differ in lifespan (*z* = −0.43, *p* = 0.67); however, control males experienced a slightly stronger relative lifespan depression in response to group living than masculinized females (*z* = 2.0, *p* = 0.045). A small difference in group living response was not surprising given the environmental contexts of the fly types differed slightly (i.e. grouped control males courted only target wild-type females, while grouped masculinized females courted each other too), as was reflected in frequency of behaviours observed, and the behaviour costs may be felt slightly different by masculinized females and wild-type males. Nonetheless, it is interesting that control males showed stronger lifespan depression despite exhibiting lower courtship rates than masculinized females (figure 2*a*,*b*). In a similar vein, feminized males also showed a more moderate reduction in life expectancy when kept in groups than control males (*z* = −3.47, *p* < 0.001; control males showed a mean lifespan reduction of 26%, while masculinized showed a reduction of 21%; figure 3*b*), despite their heightened rates of courtship. In other words, although control and feminized males both showed diminished lifespans when housed in groups compared with individuals, this reduction was proportionally smaller in feminized males. Interestingly, this difference was starkest in terms of maximum lifespan, which was reduced by 33% between control male treatments, but differed by less than 1% in feminized males, although this may be explained by the reduced variance in lifespan observed in individual feminized males.

### (b) Male-like lifespan patterns are associated with aggression and courtship receipt, not courtship display

We next sought to establish whether male lifespan patterns could be attributed more closely the expression of male sexual behaviours. To do this, in Experiment 2, we exposed masculinized and control female flies to a variety of social environments. Focal females (whose behaviour and lifespan were observed) were housed (i) individually, (ii) with wild-type females which acted as courtship targets or (iii) with masculinized females, for whom the focal fly was a courtship target (figure 1*b*).

**Figure 4.**
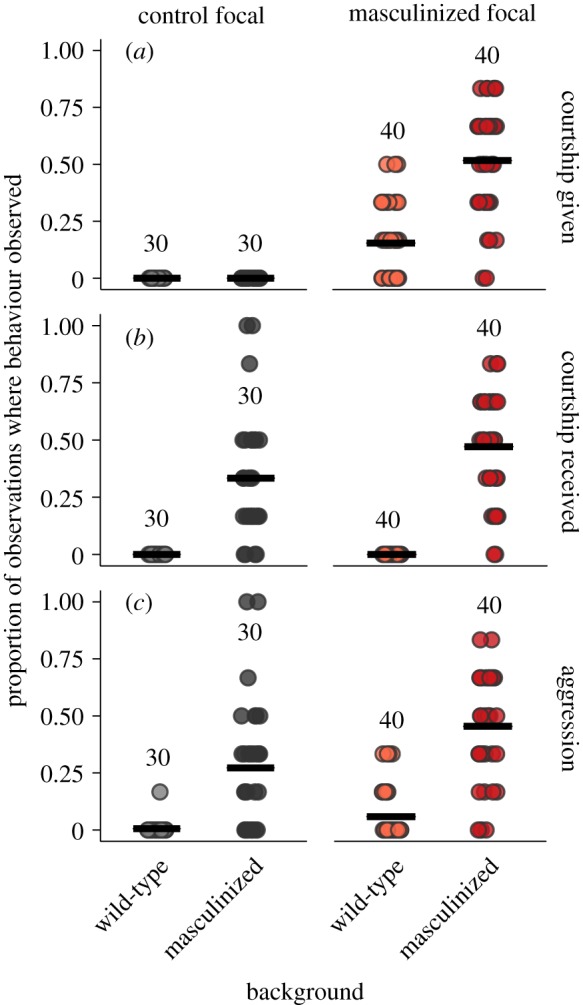
The effect of nervous system manipulation (masculinization) and social environment (masculinized versus wild-type background flies) on the proportion of observations in which behaviours involving the focal female in each vial were seen. Data points represent that cumulative proportions from nine observations are shown for behaviour types detected in each focal female. Horizontal black bars represent mean averages. Sample sizes refer to the number of vials (which is the same as the number of focal individuals). (Online version in colour.)

As expected, masculinization of the focal or background females resulted in the focal fly displaying (*t*_136_ = 4.73, *p* < 0.0001) or receiving (*t*_136_ = 7.953, *p* < 0.0001) courtship behaviour, respectively (figure 4). Surprisingly, however, there was an interaction between the effects of NS-identity and social environment on both the frequency of courtship display (*t*_136_ = 5.431, *p* < 0.0001) and courtship receipt (*t*_136_ = 1.896, *p* = 0.06) involving the focal fly, although this interaction was marginally nonsignificant in the case of courtship receipt. This was due to the fact that masculinized focal females who were housed with masculinized background females both provided and received more courtship than masculinized focals housed with wild-type background females. The cause for such elevated rates of courtship display and receipt by masculinized focals in a masculinized environment are unclear, as there is no *a priori* reason to expect that masculinized females should perceive other masculinized females as any different to wild-type females, or adjust their behaviour in response to masculinized female density separately to overall female density. However, one explanation may be because masculinized females move in search of courtship targets, and thus simply encounter other females more frequently. This behaviour is not true of control or wild type or females, so it may be the case that increasing the number of masculinized females in a vial the boosts proportion of time the focal fly spends engaged in courtship to a greater extent than would be incurred by adding control or wild-type individuals.

**Figure 5.**
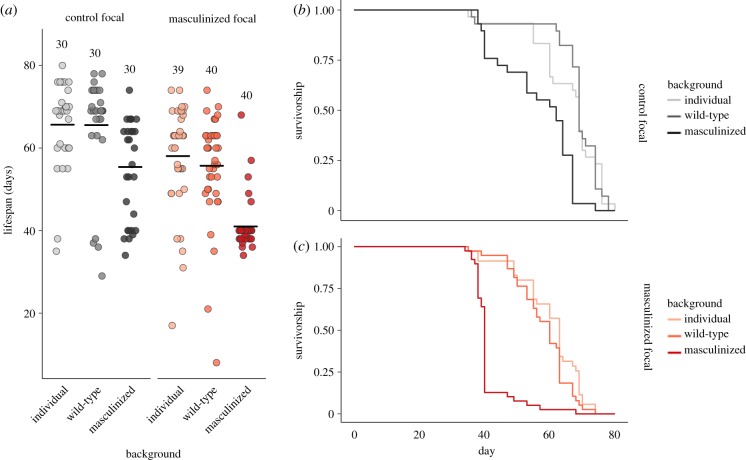
The effect of nervous system manipulation (masculinization) and social environment (individual versus group housing, with either wild-type or masculinized ‘background’ females) on lifespan for each focal female in Experiment 2. (*a*) Age at death of each focal female, with horizontal black lines representing mean averages. Sample sizes refer to the number of individual focal flies. (*b*,*c*) Survivorship curves, which give the proportion of survivors through time for each treatment. (Online version in colour.)

Although masculinized females were overall shorter lived (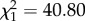, *p* < 0.0001, 14.1% difference in mean lifespan), we found no evidence of differential effects on lifespan responses between masculinized and control focal females to being housed individually, with wild-type background females, or with masculinized background females (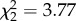, *p* = 0.15; figure 5). This lack of interaction between NS-identity and social environment indicates that displaying courtship behaviour did not have an appreciable effect on lifespan, because masculinized females did not have significantly shorter lifespans when given courting opportunities, compared to isolation. Instead, focal lifespan costs were associated with independent effects from the social environment (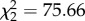, *p* < 0.0001; figure 5); specifically, focals exposed to masculinized (*z* = 8.51, *p* < 0.0001, 23.1% difference in mean lifespan), but not wild-type (*z* = 1.23, *p* = 0.22), background females experienced reduced lifespan compared with individuals. Therefore, costs probably arose from the receipt of male-specific behaviours either as recipients of courtship or through involvement in aggressive interactions.

### (c) Overall pattern indicates costs of receiving, but not expressing, male-like behaviour

It has been suggested that costs of displaying or receiving sex-specific behaviours, such as courtship, may explain sex differences in lifespan observed in nature [20,32]. Here, we manipulated sex differentiation in *D. melanogaster* to examine if there were costs to lifespan associated with behaviours encoded by sex-specific neural circuitry that are experienced even in the absence of wider sex-based physiological differences. We found that masculinized female *Drosophila* suffered reduced life expectancy when housed in groups and showed remarkably similar lifespan patterns to control males, indicating an appreciable effect of male-like behaviour on ageing. However, we found no evidence that this effect was underpinned by expression of male-like courtship, because masculinized females did not live less long when presented with courting opportunities, compared to when held in isolation. Consistent with the idea that the expression of male courtship is low cost, the lifespan-shortening effect of group living was less severe for hypercourting feminized males than for controls, i.e. the opposite of what would be expected if expressing courtship was highly costly and the main source of lifespan depression. Instead, reduced life expectancy was generally associated with the elevated *receipt* of courtship and aggression. Feminized males, who suffered a less severe cost of group living than controls, also showed less aggression. Taken together, these results indicate that observed lifespan patterns in masculinized females and feminized males were more consistent with a negative effect of receiving courtship or male-like aggression, rather with costs of expressing courtship display.

### (d) Sex-specific behaviour and life-history evolution

The potent role of the nervous system in mediating lifespan both in social and non-social situations has important implications for our understanding of sex-specific ageing. Influential work in life-history theory predicts that sexual selection suppresses life expectancy in males of promiscuous species such as *D. melanogaster* by favouring ‘high-risk, high-attrition’ strategies that prioritizes investment in to reproductive effort over long-term survival [14,16,49]. Under this hypothesis, key male-specific behaviours such as courtship display (including the active pursuit of females), which prompt energetic expenditure, would be expected to generate survival costs as per a resource allocation trade-off.

Our findings provide mixed support for this. While the similarity of behaviour and lifespan patterns in masculinized females and wild-type males appears to implicate an effect of male-like behaviour, we found that performing courtship is effectively cost-free to both males and masculinized females. However, there was no indication that our failure to identify a male cost to courtship display was because social behaviour, in general, does not affect survival, as both intrasexual aggression and receipt of male-like courtship were associated with significant shifts in lifespan patterns. Intrasexual aggression is thought to be a form of sexually selected contest behaviour and has been highlighted in both sexes as an important driver of life-history traits [32,50]. In insects, for instance, it has been shown that energetic expenditure associated with contest behaviours such as mate guarding can carry physiological costs [30,51,52]. If aggression between flies expressing male-like behaviour (i.e. males or masculinized females) *D. melanogaster* is responsible for the survival patterns we observed, our data are consistent with an important role for intrasexual selection in explaining sex differences in lifespan.

On the other hand, costs to females from receiving male-specific behaviours could arise through interlocus sexual conflict [53]. Such costs from male-induced harm, including sexual harassment, have been widely studied in *Drosophila*, as well as the effects of harmful sex peptides transferred to females via male ejaculate [54], and are thought to be particularly prevalent in driving the evolution of female life-history traits, including lifespan [14,55,56]. Male courtship behaviour *per se* is thought to have a negative effect on female fitness in *Drosophila* species [57–59] and has been shown to influence gene expression, including genes which may have downstream effects for lifespan, such as those involved with immune function and sensory processing [60,61]. If this is the case, our results may provide explicit experimental evidence of direct female lifespan costs to receipt of male-specific behaviour by showing that these costs even occur when the behaviour is received from other females. Thus, the receipt of male behaviour *per se* is costly to females and does not require other aspects of male phenotype (e.g. sight, sound or smell). While masculinized females showed reduced lifespan in response to courtship, the same was not true of hypercourting feminized males, who also received elevated rates of courtship in group treatments but showed more moderate lifespan depression than wild-type males. One interpretation of this could be that courtship receipt is only harmful to females, possibly because fitness costs are mediated through sex-specific expression of genes that are unaffected by NS manipulation.

While our experimental design allowed us to rule out costs associated with courtship display in masculinized females, fully disentangling the effects aggression and courtship receipt was beyond the scope of this study as the behaviours co-occurred across group treatments. Subsequently, we cannot determine whether the behavioural effects on lifespan we observed implicate sexually selected male costs from intrasexual competition or female costs from sexual conflict. Further work is required here to isolate courtship receipt from intrasexual aggressive behaviours (which encompasses a number of distinct interactions such as fighting and disturbance of fly foraging), and to do this it will be important to examine equivalence of the effects of masculinized female and wild-type male courtship and aggression on lifespan.

### (e) Sex-specific behaviour, social perception and other tissues

Two recent studies suggest that the costs associated with social contact sexual behaviour are mediated through perception pathways, rather than as a direct energetic cost in *D. melanogaster* [34,35]. Gendron *et al.* [34] found that flies exposed to the pheromones of the opposite sex suffered reduced lifespan and elicited key physiological responses, although the effect was far greater for males. These effects appeared independent of any behavioural differences, and, furthermore, Harvanek *et al.* [35] showed that reproductive behaviour was not itself costly for males if they did not detect female pheromones. Our findings generally support these previous studies [34,35] with regard to a negligible cost for male-like courtship display, and it is also possible that social perception was responsible for the inherent costs of masculinization in females if they were responding ‘auto-erotically’ to their own female pheromones. However, as masculinized focal females housed with wild-type females, and thus exposed to female pheromones, did not show appreciable lifespan depression, we found no evidence of perception costs in this system. Obvious reasons for this could be (i) that the physiological responses underpinning lifespan in *D. melanogaster* are mediated downstream of the nervous system and thus unaffected by masculinization, or (ii) that sex-specific olfactory or gustatory receptors prevent male-like recognition of female pheromones by masculinized females. Thus, our results do not conflict with a ‘costs of sensory perception’ hypothesis, but add to an increasingly complex story of how reproductive costs are incurred.

The apparent lack of costs to energetic behaviours such as courtship found our study and previous studies [34,35] is puzzling from the life-history trade-off theory perspective. One possibility could be that costs of behaviour are borne continuously, even if the behaviour is not expressed. Male courtship display could carry physiological costs, as predicted by life-history theory, that are actually mediated ‘constitutively’ via another system and thus undetectable to the experimental variation induced in these studies. One tissue which could act as such a platform is the gut, which has recently been implicated in patterns of sex-dependent ageing [62–64] (see [65] for review of dietary effects) and is known to have an important relationship, particularly via the microbiota, with behaviour in vertebrates (e.g. [66]) and invertebrates, including *Drosophila* (e.g. [66,67]). In this case, the developmental environment, in particular feeding conditions, could effectively mask costs associated with energetic expenditure in either sex. Interestingly, lifespan effects of dietary restriction have already been shown to be sex-specifically sensitive to variation in social environment in *D. melanogaster* [17,68], and there is evidence of similar interactions between sex, social environment and diet in the neriid fly, *Telostylinus angusticollis* [69]. Although the consequences of the microbiota–gut–brain (and behaviour) axis for lifespan have yet to be investigated, we suggest that it will be important to see how such interactions play out in future studies.

## 4. Conclusion

In this study, we used *D. melanogaster* to test for the presence of lifespan responses to sex-specific behaviour, which have been advocated as an important driver of sexual dimorphism in life expectancy [20,32]. We found that survival patterns were explained by an interaction between nervous system sexual identity and social environment, which is consistent with expectations of a behavioural basis for lifespan dimorphism. Our data indicated that male-like courtship appears surprisingly cost-free to the actor, but costly to the recipient, although it was not possible to fully disentangle effects of aggression and courtship. Thus, while our results indicate that behaviour is able to influence survival, we find little evidence that sex-specific expression of behaviours *per se*, encoded by the nervous system, mediates sex differences in lifespan.

We argue that our data are consistent with the emerging view that there is an important, but highly complex, relationship between social interactions and lifespan which appears to be mediated by a number of interdependent factors including behaviour, social perception and gut state [65]. We suggest that resolving both the effects and contingencies of these components will likely prove crucial for understanding sex differences in life expectancy, as well the synthesis of any general theoretical framework for the ageing process. Furthermore, the convoluted nature of lifespan trade-offs supports growing evidence that traditional models of life-history evolution, which view trade-offs as tractable processes directing resource distribution across independent fitness components [12], may need to be expanded to account for complexities in the proximate evolutionary mechanisms now thought to underpin life-history traits.

## Supplementary Material

Supplementary Figures

## Supplementary Material

Experiment 1 Behaviour - Females.dat

## Supplementary Material

Experiment 1 Behaviour - Males.dat

## Supplementary Material

Experiment 1 Survival.dat

## Supplementary Material

Experiment 2 Behaviour.dat

## Supplementary Material

Experiment 2 Survival.dat

## Data accessibility

The research materials supporting this publication are provided in the electronic supplementary material.
