# Supplementary material for: Interactions between the sexual identity of the nervous system and the social environment mediate lifespan in Drosophila melanogaster
Source: Proc Biol Sci. 2018 Nov 28;285(1892):20181450. doi: 10.1098/rspb.2018.1450 (PMC6283938; doi:10.1098/rspb.2018.1450)
Supplement: Supplementary Figures [file rspb20181450supp1.pdf]

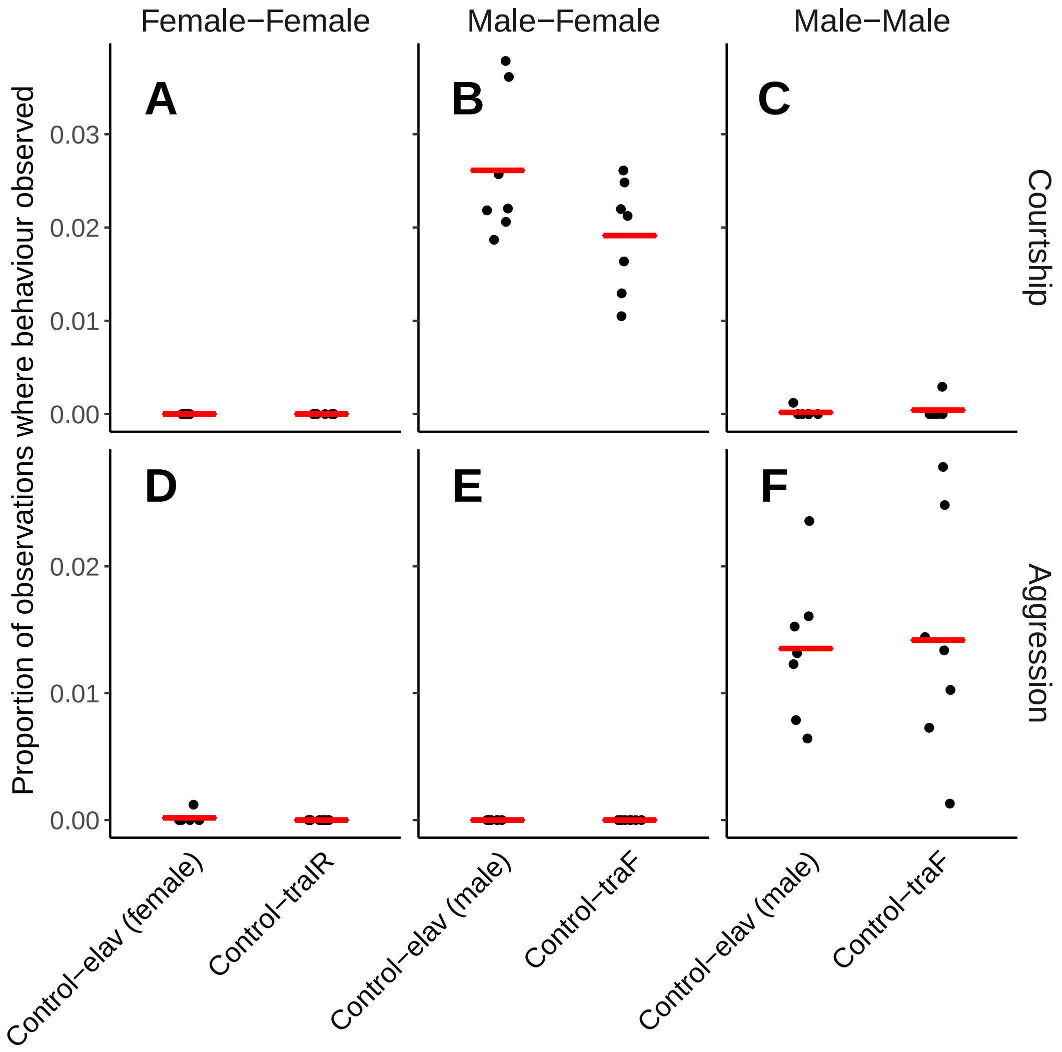

Figure 1. Observed behaviour is shown for male and female control strains in Experiment 1. Per vial cumulative proportions, mean averaged across the 10 flies in each vial, are shown for **(a-c)** observed **courtship** and **(d-f)** observed **aggression**. Female-Female and Male-Male interactions refer to intrasexual behaviour, while Male-Female interactions are intersexual behaviours performed by males on the 2 female ‘courtship target’ flies. Behaviour responses are indistinguishable between the strains, and data was pooled in to a single group.

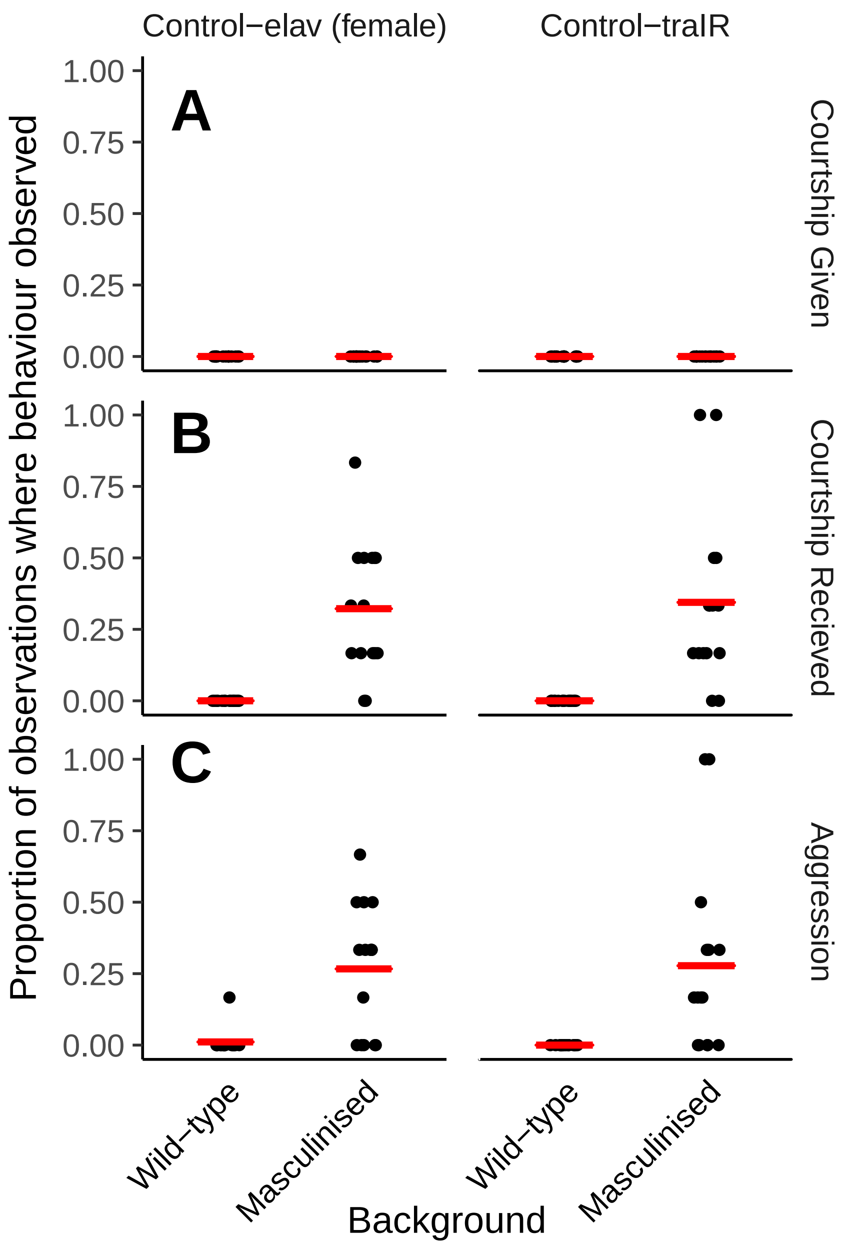

Figure 2 The effect of social environment (masculinised vs wild-type background flies) on the proportion of observations of behaviours involving a control strain focal female in each vial. Data points represent cumulative proportions from 9 observations are shown for behaviour types detected in each focal female. Behaviour responses are indistinguishable between the strains, and data was pooled in to a single group.

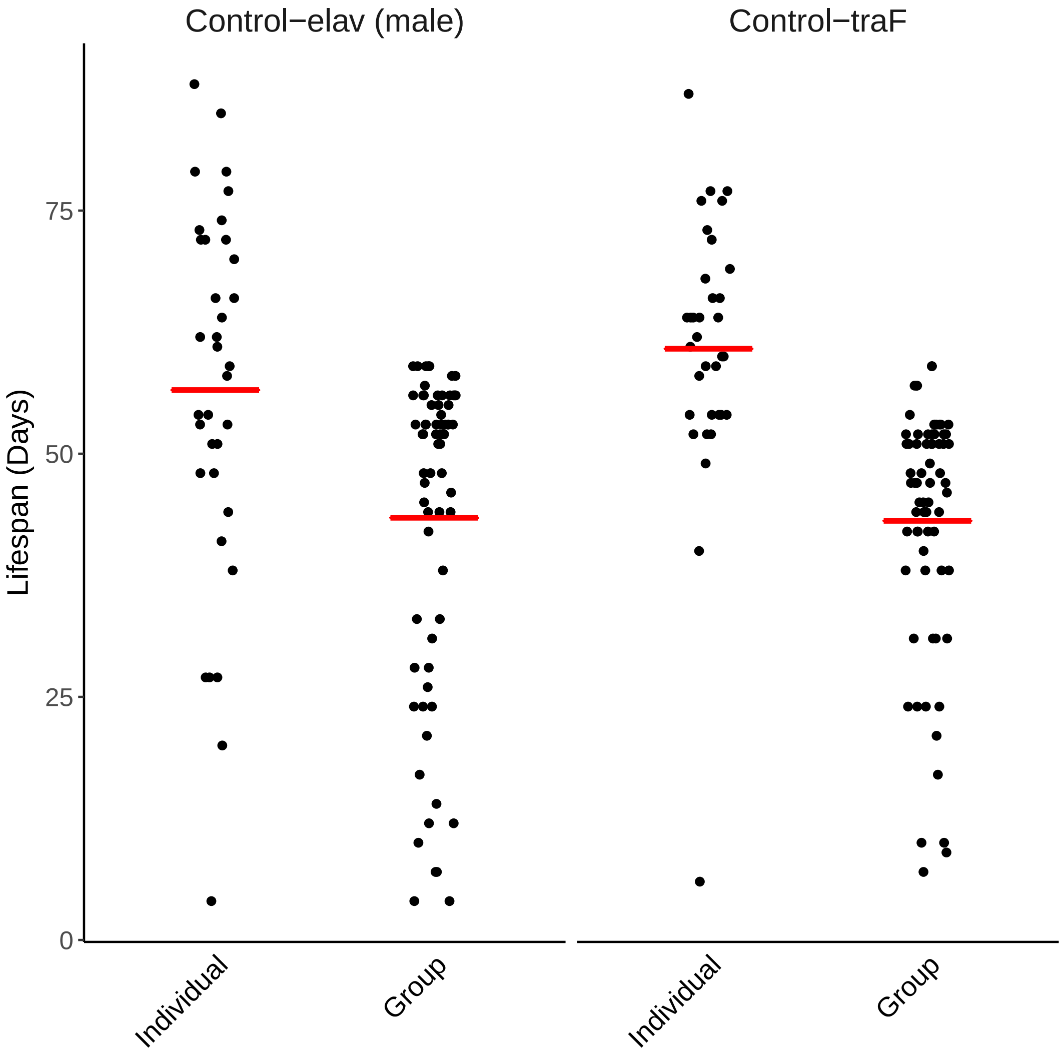

Figure 3 Lifespan under different social conditions (individual and group housing) is shown for male control strains in Experiment 1. Lifespan responses are indistinguishable between the strains, and data was pooled in to a single group.

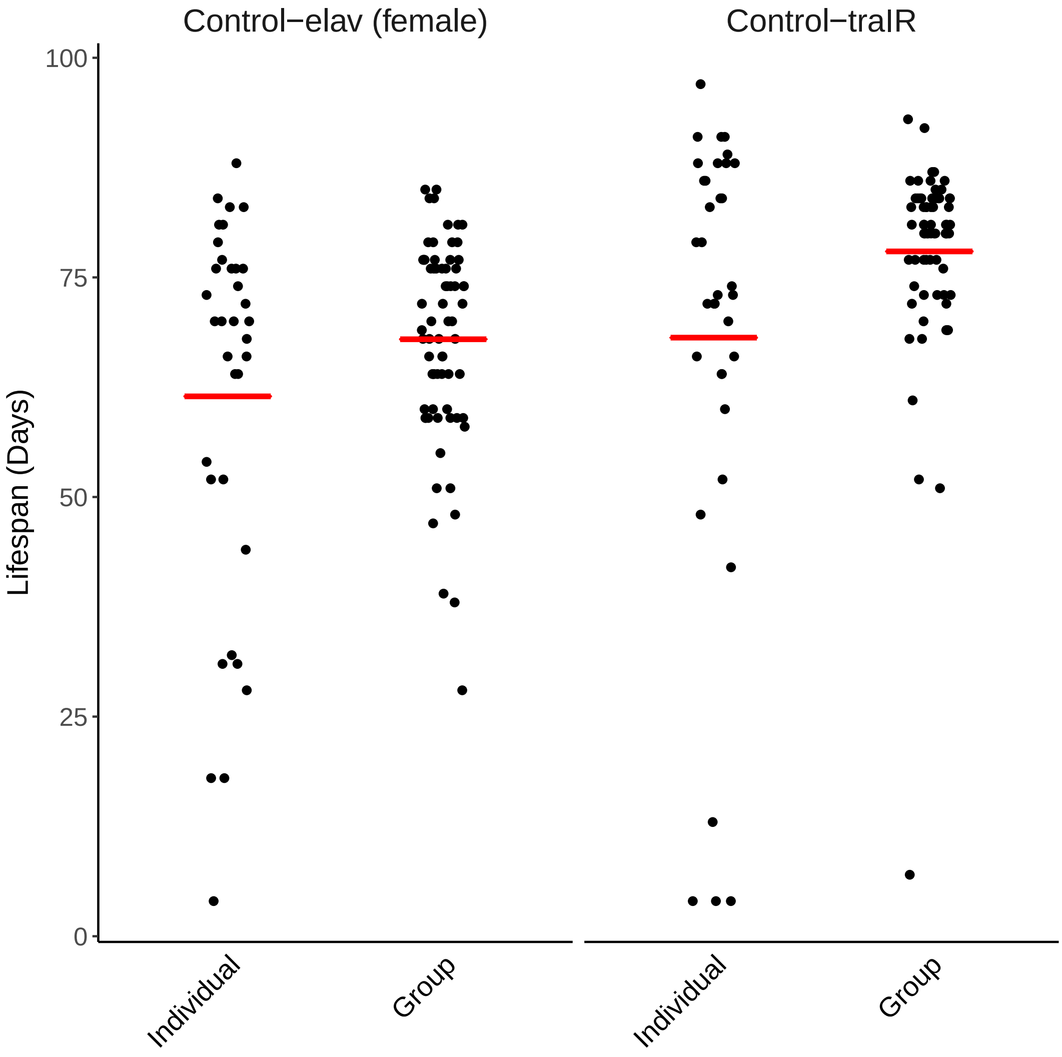

Figure 4 Lifespan under different social conditions (individual and group housing) is shown for female control strains in Experiment 1.

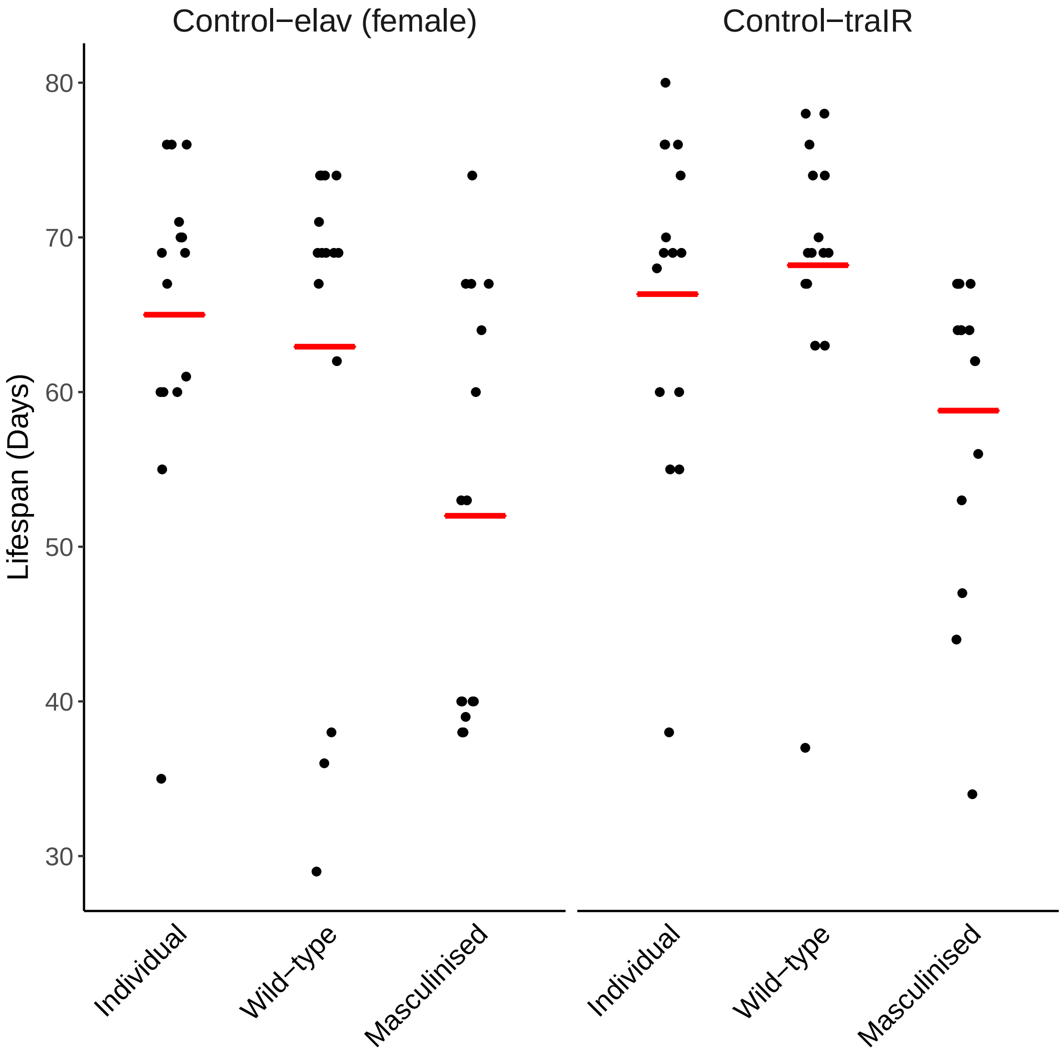

Figure 5 Lifespan under different social conditions (individual vs group housing, with either wild-type or masculinised 'background' females) is shown for female control strains in Experiment 2.

While figures 2 and 3 indicate that Control-elav females show slightly reduced lifespan compared with Control-traIR individuals, this effect appears entirely independent of social environment as responses to different housing treatments are consistent across the two strains. As a result, female control strains were also pooled.
